# Aspergillus Bronchitis at Localised Mucus Plug in an Immunocompetent Patient

**DOI:** 10.1002/rcr2.70104

**Published:** 2025-03-20

**Authors:** Kai Yamazaki, Yukihiro Horio, Kazuhito Hatanaka, Takashi Yaguchi, Kozaburo Sadahiro, Kohei Umemoto, Shigeaki Hattori, Katsuyoshi Tomomatsu, Naoki Hayama, Yoko Ito, Tsuyoshi Oguma, Koichiro Asano

**Affiliations:** ^1^ Division of Pulmonary Medicine, Department of Medicine Tokai University School of Medicine Isehara Japan; ^2^ Department of Pathology Tokai University School of Medicine Isehara Japan; ^3^ Medical Mycology Research Center Chiba University Chiba Japan

**Keywords:** Aspergillus bronchitis, *Aspergillus udagawae*, bronchiectasis, hemoptysis, mucus plug

## Abstract

Aspergillus tracheobronchitis is a form of invasive aspergillosis that primarily occurs in immunocompromised patients. We report a case of Aspergillus bronchitis in an immunocompetent 55‐year‐old woman with a mucus plug at the site of localised bronchiectasis. The mucus plug gradually enlarged over 9 years, when the patient exhibited submissive haemoptysis. Bronchial artery embolization, followed by partial lung resection was performed. Pathological and mycological examinations led to the diagnosis of AT caused by *Aspergillus udagawae*.

## Graphical abstract

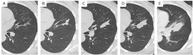

## 1 Introduction

Aspergillus tracheobronchitis (AT) is a rare form of invasive aspergillosis confined to the trachea and central bronchi, accounting for approximately 7% of all pulmonary aspergillosis cases [1]. AT mainly develops in immunocompromised patients, including those with haematologic malignancies or those receiving immunosuppressive treatments such as long‐term corticosteroid therapy or chemotherapy. Although some cases of AT develop in moderately immunocompromised patients with chronic pulmonary obstructive pulmonary disease, diabetes mellitus, or autoimmune diseases [1], there have been few reports of AT in immunocompetent patients.

Herein, we report a case of Aspergillus bronchitis that developed at the site of localised bronchiectasis and mucoid impaction in an immunocompetent woman in her 50s. The mucus plug enlarged gradually over a period of 9 years until sub‐massive haemoptysis developed.

## 2 Case Report

A 55‐year‐old Japanese woman was referred to our hospital due to a localised bronchiectasis in the lingual region of the left lung. There have been no symptoms, signs, or family history of cystic fibrosis (CF). One year later, she remained asymptomatic, however, the bronchus was filled with mucus. There was no evidence suggesting the development of allergic bronchopulmonary aspergillosis, such as peripheral blood eosinophilia, an increase in serum IgE levels, or positive *Aspergillus fumigatus* ‐specific IgE/precipitin.

**FIGURE 1:**
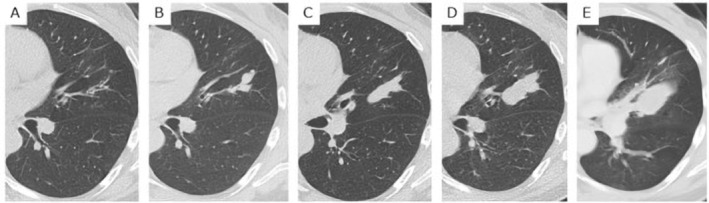
Chest computed tomography scan showed bronchiectasis in the lingual region of the left lung at the initial examination (A). One year later, a mucus plug appeared at the site of bronchiectasis (B). The mucus plug enlarged gradually at 3 years (C), 7 years (D), and 10 years (E) after the initial examination.

She had not presented with any respiratory symptoms until 6 years later, when she exhibited hemosputum. Thoracic computed tomography (CT) demonstrated enlargement of the mucus plug in the left lung (Figure 1). Bronchoscopic examination showed no abnormality in the bronchi except for mucous sputum. Bronchial lavage showed negative culture for bacteria and fungi, although sputum culture was positive for *Cladosporium* sp. and *Penicillium* sp.

**FIGURE 2:**
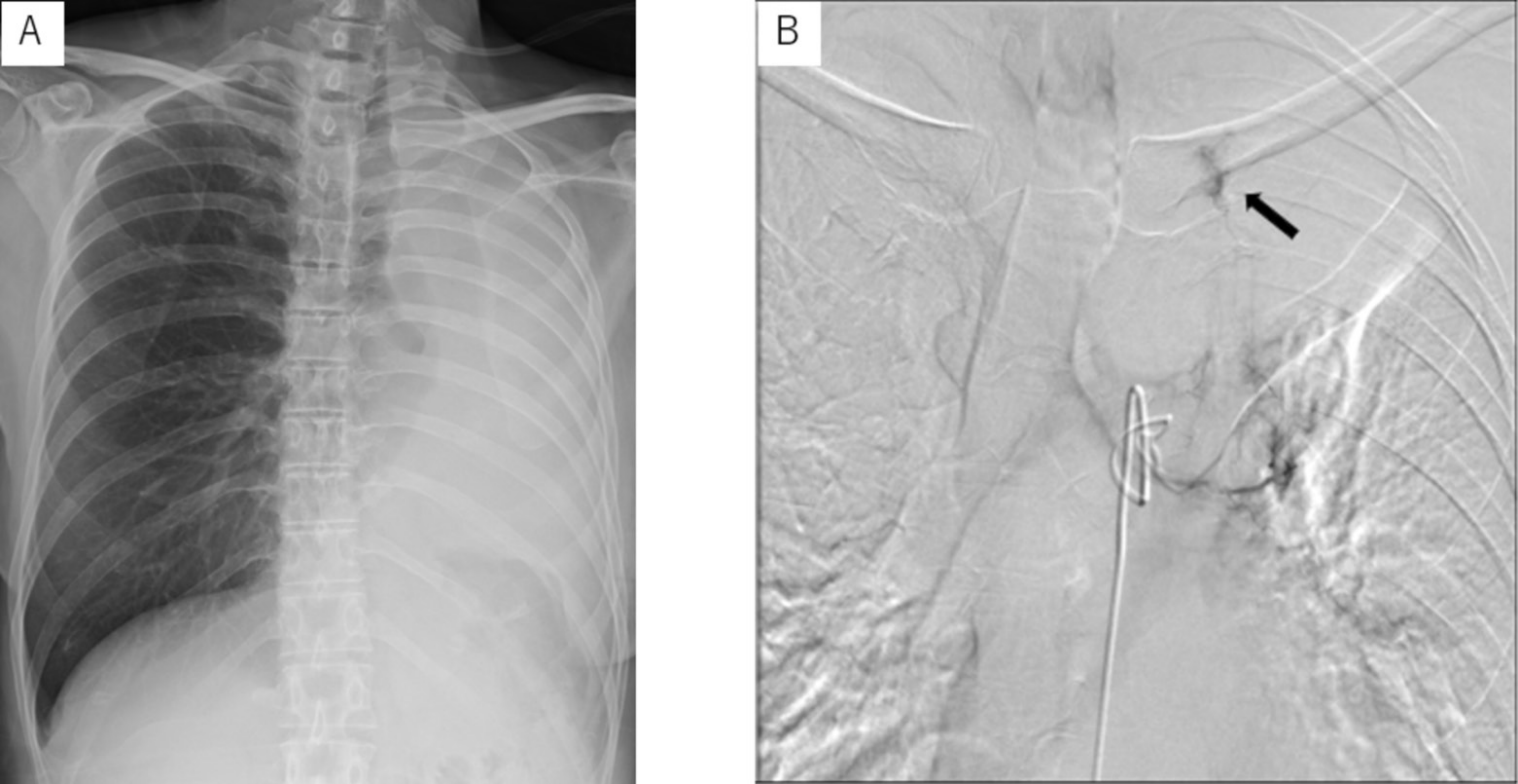
(A) x‐radiograph showed complete atelectasis of the left lung. (B) Angiography of left bronchial artery. A ring‐shaped dark staining surrounding the mucus plug was observed in the lingual region of the left lung.

Three years after the first episode of hemosputum, she was admitted to the hospital due to sub‐massive hemoptysis. Contrast‐enhanced CT scan of the chest showed atelectasis of the left lung accompanied by an enlarged mucous plug in the lingual region. Angiography of the left bronchial artery showed a staining in the legion compatible with the collapsed lingual area; bronchial artery embolization was performed with absorbable gelatin sponge (Figure 2). After embolization, the hemoptysis disappeared and the atelectasis in the left upper lobe was improved. She was discharged on the 16th day.

**FIGURE 3:**
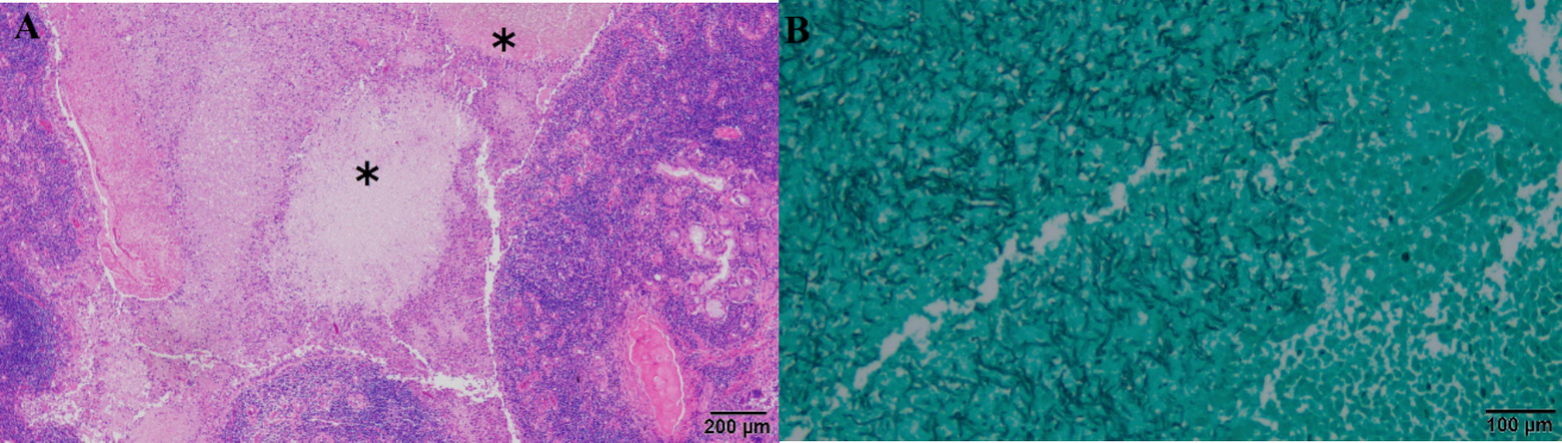
Histopathology of the specimen surgically resected from the lingual region of the left lung. (A) Haematoxylin‐eosin staining (20×). The bronchial lumen was filled with fungal hyphae (*) and the wall was infiltrated with inflammatory cells, mainly neutrophils. (B) Grocott staining (100×) showed Y‐shaped hyphae in the bronchial lumen.

A left lingual segmentectomy was performed 7 months after the discharge to avoid the recurrent hemoptysis due to residual lesions. Histopathological examination of the surgical specimen revealed fungal hyphae filling the lumen of the bronchi, which wall was infiltrated with inflammatory cells, mostly neutrophils (Figure 3). Fungal hyphae were found in some pulmonary arteries, although there was no evidence of embolization or necrosis. Culture of the lung specimen showed *A. udagawae*, which was identified by genetic analysis of calpain gene. There was no recurrence of hemoptysis for 10 months since the surgery.

## 3 Discussion

Bronchopulmonary aspergillosis can take three forms according to the status of the immune system and preexisting pathology in the lungs: allergic disease, non‐invasive infection, and invasive infection [2]. In the peripheral lungs, chronic pulmonary aspergillosis (CPA) develops as non‐invasive infection in immunocompetent hosts with anatomical anomaly such as cavity, whereas invasive pulmonary aspergillosis develops as invasive infection in immunocompromised hosts. In the proximal airways, ABPA, which develops as a hypersensitivity reaction to fungi colonising the airways, and AT due to invasive infection of the trachea and bronchi [1] are the major forms of aspergillosis. In the present case, ABPA was suspected because of the presence of an intrabronchial mucus plug and central bronchiectasis in an immunocompetent patient. However, the patient lacked clinical presentation and pathology required for the diagnosis of ABPA, such as *A. fumigatus* ‐specific IgE and eosinophilic mucus plugs with Charcot‐Leyden crystals.

Active fungal growth was observed in the present case, not only in the bronchial lumen but also in the pulmonary artery of the resected lung specimen, suggesting AT. Despite intensive antifungal treatment, the mortality rate of AT is as high as 39.1%, particularly in patients with neutropenia, acute renal failure, or those presenting with acute respiratory failure and fever at the time of diagnosis [1]. Even in patients who are not severely immunocompromised, mortality is as high as 23.7% [3]. On the contrary, this case developed Aspergillus bronchitis in a pre‐formed intrabronchial mucus plug in an immunocompetent host and was associated with a chronic course, equivalent to CPA in the peripheral lung. Once *Aspergillus* spp. colonised the pre‐formed mucus plug, the fungi could not be removed by the mucociliary clearance system, establishing a non‐invasive infection. Similar cases of chronic Aspergillus bronchitis have been reported in CF patients characterised by tenacious mucus in the bronchi [2, 4]. However, chronic Aspergillus bronchitis is rare in non‐CF cases, and furthermore, this case eventually developed sub‐massive hemoptysis with fungal invasion into the pulmonary vessels, not reported in the cases of CF‐associated chronic disease.

In the present case, surgical resection was selected, and additional antifungal treatment was not performed because there were no residual lesions in the lungs, and the causative fungus, *A. udagawae*, tended to be less susceptible to antifungal agents [5]. Although there have been no reports of *A. udagawae* or other cryptic *Aspergillus* species causing Aspergillus (tracheo)bronchitis, these species are increasingly recognised as pathogens in human diseases. Proper identification of these species is important for the appropriate treatment strategy.

In conclusion, a non‐invasive type of Aspergillus bronchitis can develop in the mucus plugs of immunocompetent hosts. Surgical resection is an option if the lesion is localised.

## Author Contributions

Kai Yamazaki and Yukihiro Horio drafted the manuscript. Kozaburo Sadahiro, Kohei Umemoto, Shigeaki Hattori, and Katsuyoshi Tomomatsu collected the clinical data. Kazuhito Hatanaka performed the pathological analysis of the lungs, and Takashi Yaguchi performed the genetic analysis of the isolated fungus. Naoki Hayama, Yoko Ito, Tsuyoshi Oguma, and Koichiro Asano revised the manuscript critically. All authors approved the final manuscript.

## Ethics Statement

The authors declare that appropriate written informed consent was obtained for the publication of this manuscript and accompanying images.

## Conflicts of Interest

The authors declare no conflicts of interest.

## Data Availability

The data that support the findings of this study are available from the corresponding author upon reasonable request.
